# Spontaneous Anterior Arch Fracture of the Atlas Following C1 Laminectomy in a Patient With Osteopetrosis: A Case With Five Years of Follow-Up

**DOI:** 10.7759/cureus.79471

**Published:** 2025-02-22

**Authors:** Yoshihiro Ishihama, Terumasa Ikeda, Shunki Iemura, Kensuke Toriumi, Koji Goto

**Affiliations:** 1 Orthopedic Surgery, Kindai University Hospital, Osakasayama, JPN; 2 Orthopedics, Kindai University Hospital, Osakasayama, JPN

**Keywords:** anterior arch fracture of the atlas, c1 laminectomy, case report, osteopetrosis, spontaneous fracture

## Abstract

Osteopetrosis is a rare group of genetic disorders characterized by excessive bone density due to impaired osteoclast function and can lead to various complications, including fractures and immune dysfunction. We describe the case of a 63-year-old man with osteopetrosis who presented with cervical discomfort and was diagnosed with an anterior arch fracture of the atlas, a rare spontaneous fracture following C1 laminectomy. Initially, no neurological abnormalities were observed, and imaging confirmed the continuity of the transverse ligament. Thus, the decision was made to follow the natural course in the absence of instability. Conservative treatment with a cervical collar was initiated, and the fracture gap began to fill by three years postoperatively, though nonunion persisted at five years. The patient remained asymptomatic and did not require further surgical intervention.

This case highlights that stress concentrated on the anterior arch of the atlas due to the unique bone quality in osteopetrosis, and the subsequent natural course of the fracture did not require additional surgical treatment. Careful follow-up is essential to monitor for any pathological changes.

## Introduction

Osteopetrosis is a rare disorder characterized by osteoclast dysfunction and impaired bone resorption [1,2], leading to bone fragility and an increased risk of fractures. Because of the reduced bone resorption by osteoclasts, bones become excessively dense and brittle [3]. Both bone quantity and quality suffer because bone formation does not progress as a result of impaired bone resorption. The resulting bone fragility makes fracture a frequent problem for patients with osteopetrosis. While limb fractures are common in osteopetrosis, spinal fractures are relatively rare [4].

To our knowledge, there have been no reported cases of anterior atlas fracture following C1 laminectomy in patients with osteopetrosis. Atlas fractures account for 2%-13% of traumatic injuries to the cervical spine and 1%-2% of all spinal injuries [5,6]. These fractures, typically resulting from axial loading or hyperextension of the cervical spine, were first reported by Jefferson [7]. While these fractures commonly occur due to trauma, they can also occur following C1 laminectomy performed for various pathologies such as spinal cord meningioma, retro-odontoid pseudotumor, atlantoaxial subluxation, cerebellar astrocytoma, cervical spondylotic myelopathy [8]. This report presents the outcome of five years of the natural course of a spontaneous anterior arch fracture of the atlas following C1 laminectomy in a patient with osteopetrosis, along with a literature review.

## Case presentation

A 63-year-old man presented with a complaint of clumsiness in both hands, which was attributed to atlantoaxial subluxation and cervical spondylotic myelopathy. He has dental malformation, and the imaging findings showed diffuse bone sclerosis, sclerosis of the cranial base, and sandwich vertebrae. Having identified the CLCN7 mutation, he was diagnosed with osteopetrosis. He had no previous fractures due to osteopetrosis. Physical examination revealed muscle weakness in the right deltoid and in the abductor digiti minimi bilaterally, limited finger movement, difficulty with tandem gait, and urinary dysfunction. Reflexes were hypoactive in the upper extremities and hyperactive in the lower limbs.

**Figure 1:**
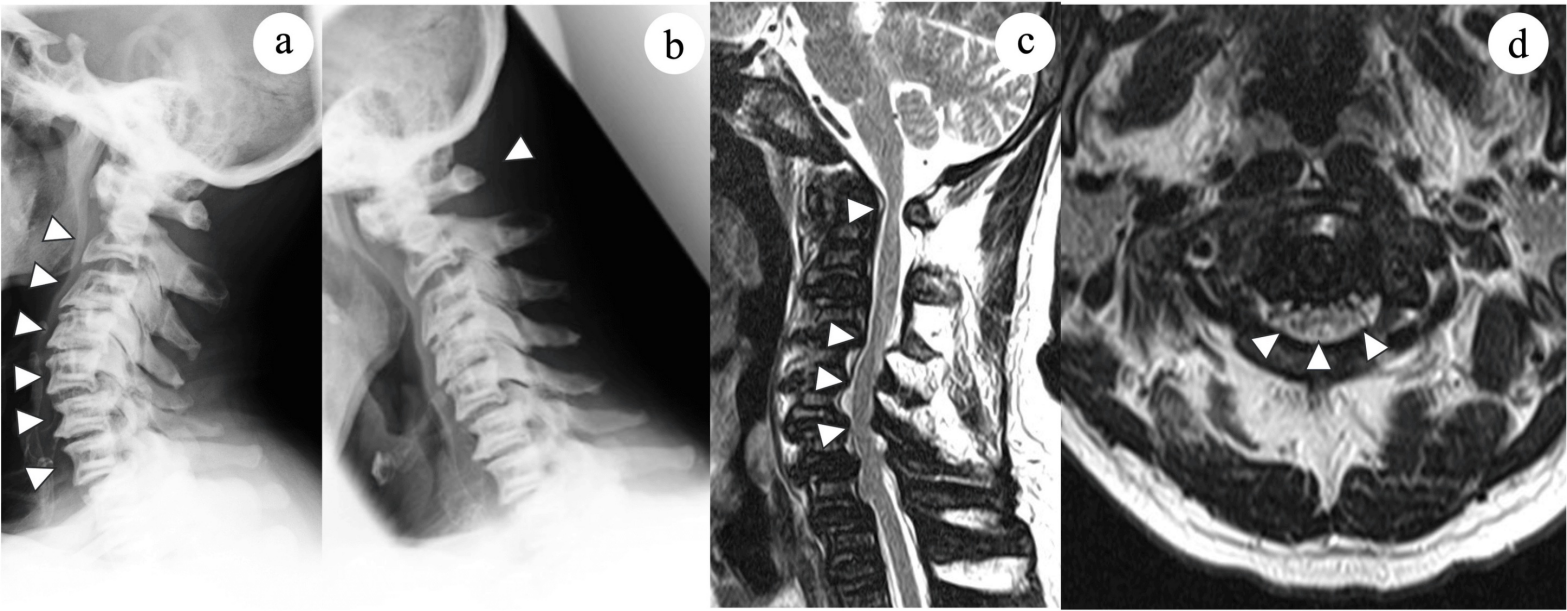
Radiological findings before surgery. (a, b) Plain radiographs showing sclerosis of the vertebral endplates and atlantoaxial subluxation (arrowheads). (c) Sagittal and (d) axial T2-weighted magnetic resonance images reveal spinal cord compression at C1 and C4–6 (arrowheads) with the bone marrow space showing a lack of signal alternating with a signal pattern similar to that of the intervertebral discs, giving a “stepladder” appearance.

Plain radiographs showed chalk-like bones and a spine with a “rugger jersey” appearance, indicating increased density at the endplates with relative lucency in the central vertebral body (Figure 1a). The atlas-dens interval was 6.68 mm, indicating atlantoaxial subluxation, with 12.4 mm available for the spinal cord (Figure 1b). Magnetic resonance imaging revealed a reduction of the bone marrow space and spinal stenosis at C1-2 and C4-6 (Figures 1c, 1d).

**Figure 2:**
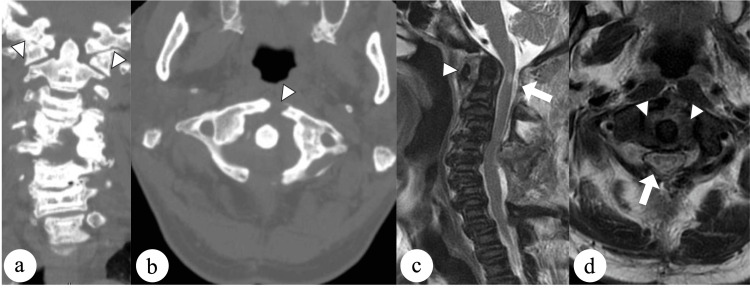
Radiological findings at one week after surgery. (a) Coronal and (b) axial computed tomography scans showing a displaced fracture of the anterior arch with widening of less than 7 mm (arrowheads). (c) Sagittal and (d) axial magnetic resonance images showing hemorrhage around the fracture site with no injury to the transverse atlantal ligament (arrowheads). Adequate direct decompression was obtained by C1 laminectomy (arrows).

The patient underwent laminectomy at C1 and C4-C6. However, several days after the surgery, he reported neck discomfort. Follow-up computed tomography scans obtained 1 week after surgery showed an atraumatic anterior arch fracture of the atlas (Figures 2a, 2b). T2-weighted magnetic resonance images confirmed hemorrhage around the fracture site but no injury to the transverse atlantal ligament (Figures 2c, 2d).

**Figure 3:**
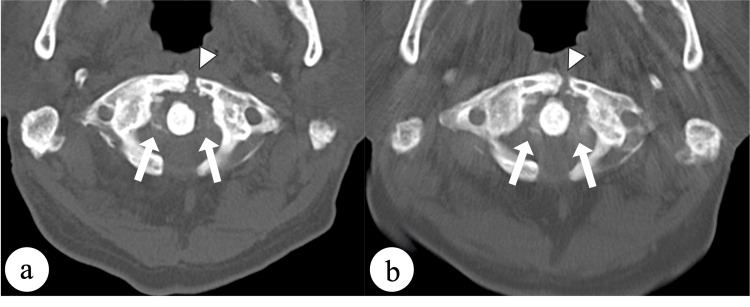
Findings on follow-up axial computed tomography scans. (a) Image obtained at three years postoperatively showing filling of the fracture gap (arrowhead) and calcification of the transverse ligament (arrows). (b) Image obtained five years postoperatively showing persistence of nonunion (arrowhead) and progressive calcification of the transverse ligament (arrows).

Conservative treatment with a Philadelphia cervical collar was initiated in view of the unique bone quality problem associated with osteopetrosis, which posed a risk for screw fixation, and the absence of significant instability. Pseudoarthrosis was diagnosed at a follow-up one year later, but orthotic treatment was discontinued because of the absence of dynamic instability. Although the fracture gap started to fill by three years postoperatively, nonunion persisted for five years. The patient has remained asymptomatic and not require further surgical intervention (Figures 3a, 3b).

## Discussion

Osteopetrosis is a hereditary disorder first identified by Albers-Schonberg in 1904 [9]. An epidemiological study in Denmark reported the prevalence of osteopetrosis as 5.5 per 100,000 population [10], indicating that it is a rare disease. Several phenotypes of osteopetrosis have been described, distinguished by the pattern of inheritance, which can be autosomal dominant, autosomal recessive, or X-linked [11]. Osteopetrosis is also classified into malignant, benign, and intermediate forms based on clinical severity, which is associated with the hereditary type [12]. The phenotype of osteopetrosis in the current case was the benign adult autosomal dominant form, and there was no history of obvious pathological fractures.

To our knowledge, there have been no other reports of spontaneous anterior arch fractures of the atlas following C1 laminectomy in a patient with osteopetrosis. In this condition, impaired osteoclast activity leads to bone fragility and increased fracture risk. Typically, fractures in osteopetrosis are transverse and perpendicular to the cortical bone surface [13]. In this case, an atraumatic fracture occurred in the anterior arch of C1, also perpendicular to the cortical bone. Postoperative use of a cervical soft collar did not prevent fracture in this case. Although this C1 fracture could not be predicted before laminectomy, we used low-speed drilling, spaced cycles, cold saline irrigation, and frequent drill bit changes to minimize the risk of intraoperative fractures resulting from the unique bone morphology.

There have been reports of anterior arch fracture following C1 laminectomy in patients without osteopetrosis. Shimizu et al. demonstrated that an abnormal concentration of von Mises stress in the anterior arch, especially when there is a defect in the posterior arch, can lead to spontaneous fracture [14]. This mechanism may explain the fracture pattern in osteopetrosis. A large inferior facet angle (greater than 23.0°) and subaxial ankylosis are independent risk factors for anterior arch fracture [15], suggesting the need for computed tomography examination before surgery. The concentration of stress may increase the risk of anterior arch fracture after C1 laminectomy in patients with osteopetrosis to a level higher than that in the general population.

The treatment of fractures in osteopetrosis remains controversial due to the lack of definitive therapeutic guidelines. Conservative treatments for cervical and lumbar disorders associated with osteopetrosis have been more frequently reported, whereas surgical treatments remain scarce [16]. Rathod et al. reported multiple fractures and unifacetal dislocation of the cervical spine in osteopetrosis, with fractures healing without residual instability or symptoms after six weeks of continuous traction, followed by six weeks of hard cervical collar treatment [17]. Fracture healing in osteopetrosis is generally either normal or delayed, with healing time typically prolonged, especially in adults [18]. In this case, conservative treatment was initiated due to the distinctive bone quality problem linked with osteopetrosis, which presented a risk for screw fixation, along with the absence of significant instability. Regarding instability, CT scans revealed a displaced fracture of the anterior arch with a widening of less than 7 mm. Sagittal and axial magnetic resonance images showed the fracture site with no injury to the transverse atlantal ligament. Given these findings, conservative management was selected following the AO Spine treatment guidelines. Although pseudoarthrosis was observed, no pathological changes were detected during five years of follow-up after surgery, and no additional surgical intervention was required. If neurological symptoms or increased instability arise in the future, additional surgery may become necessary. Surgical stabilization has shown moderate success in unstable fractures [19]. However, challenges in cervical spine instrumentation have been noted in osteopetrosis, such as iatrogenic fractures at the donor site, difficulties with hand drilling and tapping, and potential fractures around screw holes [20]. Given these challenges in spinal surgery for osteopetrosis, further studies are needed to determine the optimal treatment for this rare disease.

## Conclusions

This case highlights that stress concentrates on the anterior arch of the atlas due to the unique bone quality in osteopetrosis following C1 laminectomy. Despite the pseudoarthrosis observed in this patient, the intact transverse ligament provided stability during five years of follow-up after surgery, and no additional surgical intervention was needed. However, follow-up is continuing to monitor for any pathological changes.
